# Analysis of factors affecting nonalcoholic fatty liver disease in Chinese steel workers and risk assessment studies

**DOI:** 10.1186/s12944-023-01886-0

**Published:** 2023-08-09

**Authors:** Rui Meng, Hui Wang, Zhikang Si, Xuelin Wang, Zekun Zhao, Haipeng Lu, Yizhan Zheng, Jiaqi Chen, Huan Wang, Jiaqi Hu, Ling Xue, Xiaoming Li, Jian Sun, Jianhui Wu

**Affiliations:** School of Public Health, North China University of Science and Technology, Caofeidian New Town, No. 21 Bohai Avenue, Tangshan, 063210 China

**Keywords:** Steel workers, NAFLD, Influencing factors, Risk assessment, Risk scoring system

## Abstract

**Background:**

The global incidence of nonalcoholic fatty liver disease (NAFLD) is rapidly escalating, positioning it as a principal public health challenge with significant implications for population well-being. Given its status as a cornerstone of China's economic structure, the steel industry employs a substantial workforce, consequently bringing associated health issues under increasing scrutiny. Establishing a risk assessment model for NAFLD within steelworkers aids in disease risk stratification among this demographic, thereby facilitating early intervention measures to protect the health of this significant populace.

**Methods:**

Use of cross-sectional studies. A total of 3328 steelworkers who underwent occupational health evaluations between January and September 2017 were included in this study. Hepatic steatosis was uniformly diagnosed via abdominal ultrasound. Influential factors were pinpointed using chi-square (χ^2^) tests and unconditional logistic regression analysis, with model inclusion variables identified by pertinent literature. Assessment models encompassing logistic regression, random forest, and XGBoost were constructed, and their effectiveness was juxtaposed in terms of accuracy, area under the curve (AUC), and F1 score. Subsequently, a scoring system for NAFLD risk was established, premised on the optimal model.

**Results:**

The findings indicated that sex, overweight, obesity, hyperuricemia, dyslipidemia, occupational dust exposure, and ALT serve as risk factors for NAFLD in steelworkers, with corresponding odds ratios (OR, 95% confidence interval (CI)) of 0.672 (0.487–0.928), 4.971 (3.981–6.207), 16.887 (12.99–21.953), 2.124 (1.77–2.548), 2.315 (1.63–3.288), 1.254 (1.014–1.551), and 3.629 (2.705–4.869), respectively. The sensitivity of the three models was reported as 0.607, 0.680 and 0.564, respectively, while the precision was 0.708, 0.643, and 0.701, respectively. The AUC measurements were 0.839, 0.839, and 0.832, and the Brier scores were 0.150, 0.153, and 0.155, respectively. The F1 score results were 0.654, 0.661, and 0.625, with log loss measures at 0.460, 0.661, and 0.564, respectively. *R*^*2*^ values were reported as 0.789, 0.771, and 0.778, respectively. Performance was comparable across all three models, with no significant differences observed. The NAFLD risk score system exhibited exceptional risk detection capabilities with an established cutoff value of 86.

**Conclusions:**

The study identified sex, BMI, dyslipidemia, hyperuricemia, occupational dust exposure, and ALT as significant risk factors for NAFLD among steelworkers. The traditional logistic regression model proved equally effective as the random forest and XGBoost models in assessing NAFLD risk. The optimal cutoff value for risk assessment was determined to be 86. This study provides clinicians with a visually accessible risk stratification approach to gauge the propensity for NAFLD in steelworkers, thereby aiding early identification and intervention among those at risk.

**Supplementary Information:**

The online version contains supplementary material available at 10.1186/s12944-023-01886-0.

## Background

NAFLD represents a form of liver injury attributable to metabolic stress, characterized by excess fat accumulation in hepatocytes, in the absence of excessive alcohol consumption or other evident hepatotoxic factors. NAFLD encompasses a spectrum of conditions, including hepatocellular carcinoma, liver cirrhosis, nonalcoholic steatohepatitis, and nonalcoholic hepatic steatosis, all of which exert a significant health impact on the population [1].

Recent research indicates that the global prevalence of NAFLD is considerably higher than previously estimated and is increasing at a worrisome rate. Prior to 2005, the prevalence of NAFLD stood at a significant 37.8%, but from 2016 onward, the figure increased even further, bringing the overall global prevalence to an alarming 32.4% [2]. In the United States alone, NAFLD affects over 80 million individuals, whereas in Asia, the total prevalence is estimated to be as high as 27.4% [3]. Furthermore, the incidence of NAFLD is not confined to the middle-aged population but extends to children and adolescents as well [4, 5]. The prevalence of NAFLD/NASH (nonalcoholic steatohepatitis) has been rising at an annual rate of 1.35%, causing a surge from 19.34 million cases in 1990 to 29.49 million in 2017 in children and adolescents [5]. NAFLD has indeed emerged as a significant public health issue of global concern.

The pathogenesis of NAFLD is multifaceted, and a variety of theories have been proposed to explain it. The traditional 'two-hit' theory posits that insulin resistance (IR) initiates a 'first hit' to the liver by triggering fat accumulation in hepatocytes, followed by a 'second hit' due to oxidative stress incited by reactive oxygen species (ROS), thereby promoting liver disease [6]. However, it soon became evident that the progression of NAFLD was not solely dictated by this 'second hit,' but instead by multiple parallel factors in genetically predisposed individuals operating synergistically. As a result, the 'multiple-hit' hypothesis was formulated [7]. According to this hypothesis, an array of factors, including dietary habits, environmental influences, obesity, and genetic predispositions, all contribute to the onset of NAFLD, thus providing a more comprehensive foundation for NAFLD management.

As a cornerstone of the Chinese economy, the steel industry employs a significant number of workers, rendering occupational health studies crucial. Steelworkers are routinely exposed to occupational hazards such as shift work, elevated temperatures, noise, and dust. Consequently, compared to the general population, they exhibit higher rates of obesity and hypertension [8]. Research has established a direct correlation between metabolic disorders such as obesity, dyslipidemia, and NAFLD [9]. It was also discerned that night shift work further exacerbated NAFLD in steelworkers [10]. These findings imply that steelworkers may face an elevated risk of NAFLD. Therefore, executing a risk assessment for NAFLD in steelworkers bears significant practical implications. This would not only enhance the health status of those working in the steel industry but also facilitate the enactment of comprehensive prevention and treatment programs within steel mills.

In the era of big data, machine learning techniques have seen rapid advancements, offering innovative technical tools for disease risk assessment, including NAFLD. This study aims to develop a methodology for evaluating the risk of NAFLD in steelworkers by employing logistic regression, random forest, and XGBoost algorithms. The best-performing model will be selected to guide further exploration and investigation into factors associated with NAFLD in steelworkers.

## Methods

### Study Subject

The current investigation is a cross-sectional study that analyzed baseline data collected between January and September 2017. These data were sourced from the National Key Research and Development Program under the project entitled "The Beijing-Tianjin-Hebei Regional Occupational Population Health Effects Cohort Study". In total, 3328 individuals were included in the study. The inclusion criteria were as follows: age ≤ 60, regular employees with a service length of at least one year, and voluntary participation with a signed informed consent form. The exclusion criteria were as follows: excessive alcohol intake (> 210 g/week for men and 140 g/week for women), severe liver disease (including acute and chronic hepatitis, viral hepatitis, cirrhosis, etc.), incomplete information, and loss to follow-up.

### Collection of information

Data were gathered via questionnaire surveys administered through one-on-one interviews conducted by expertly trained master's and doctoral students from the School of Public Health of North China University of Technology. The survey encompassed a broad range of topics, including demographic information (age, sex, ethnicity, marital status, education level, economic income), lifestyle behaviors (smoking, alcohol consumption, dietary habits, physical activity), personal and family disease history (hypertension, diabetes, and other family medical history), and occupational information (length of service, shift work, exposure to harmful occupational factors).

### Laboratory tests

Each day, before 9:00 a.m., fasting blood samples and morning urine specimens were collected by a medical examination hospital and dispatched to the laboratory for analysis. Assessed parameters included fasting plasma glucose (FPG), uric acid (UA), total cholesterol (TC), triglyceride (TG), high-density lipoprotein cholesterol (HDL-C), low-density lipoprotein cholesterol (LDL-C), alanine aminotransferase (ALT), aspartate aminotransferase (AST), glutamyl transpeptidase (GGT) and total bilirubin (TBil). All biochemical analyses of blood samples were conducted utilizing the Mindray automatic biochemical analyzer (BS-800).

### NAFLD diagnostic criteria

The diagnosis of hepatic steatosis was consistently determined by abdominal ultrasound from ultrasonographers at the examining hospital who were unaware of the purpose of this study or the subjects' exposure. A high-resolution B-mode topographic ultrasound system (PHILIPS, HD7, China) was used for diagnosis.

The presence of fatty liver was confirmed when any two of the following three ultrasound findings were observed [11]: (1) Diffusely enhanced near-field echogenicity of the liver (termed 'bright liver'), demonstrating greater echogenicity than the kidneys. (2) Poorly visualized intrahepatic ductal structures. (3) Gradual attenuation of the liver's far-field echogenicity. The final diagnosis of NAFLD excluded excessive alcohol consumption, the influence of relevant medications (such as acetaminophen, methotrexate, tamoxifen or glucocorticoids), and specific liver diseases known to induce hepatic steatosis (for instance, hepatitis C virus infection, cirrhotic degeneration, or autoimmune hepatitis) [1].

### Variable definition

#### Diabetes [12]

Defined as fasting blood glucose (FPG) ≥ 7.0 mmol/L or a previously diagnosed condition with ongoing diabetes treatment.

#### Hypertension [13]

Characterized by a systolic blood pressure (SBP) ≥ 140 mmHg and/or diastolic blood pressure (DBP) ≥ 90 mmHg or a previously diagnosed condition with current hypertension management.

#### Dyslipidemia [14]

Marked by a TC ≥ 6.2 mmol/L (240 mg/dL), TG ≥ 2.3 mmol/L (200 mg/dL), LDL-C ≥ 4.1 mmol/L (160 mg/dL), HDL-C < 1.0 mmol/L (40 mg/dL), or previously diagnosed hyperlipidemia with ongoing lipid-lowering medication.

#### Physical activity [15]

Categorized as mild, moderate or severe physical activity as per the International Physical Activity Questionnaire (IPAQ).

#### Body mass index

BMI = weight (kg)/height^2^ (m^2^). The Chinese Adult Weight Determination Standard (WS/T 428–2013) defines 24.0 kg/m^2^ ≤ BMI < 28.0 kg/m^2^ as overweight and BMI ≥ 28.0 kg/m^2^ as obese.

#### Diet [16]

Assessed based on the consumption of whole grains, vegetables, fruits, low-fat milk, nuts and legumes, sugary drinks, red meat and processed meat products, and sodium intake. Dietary scores were computed as per the Dietary Approaches to Stop Hypertension (DASH). In this study, the median NASH score was 25, with dietary profiles segmented into DASH < 25 and DASH ≥ 25, where a higher score indicates a healthier diet.

#### Hyperuricemia [17]

Classified as blood uric acid ≥ 420 μmol/L in men and 360 μmol/L in women or a history of treated gout.

#### Smoking

Defined as per the WHO's 1997 classification of smoking as the consumption of at least one cigarette per day for a duration exceeding six months. In this study, smoking status was categorized as never smoked, quit smoking, or currently smoking.

#### Shift work

Described as a working hour system where hours are variable, involving one or several groups working in shifts for continuous 24-h operation. Categories include never-shift work, once-shift work, and now-shift work.

#### Occupation dust [18]

Determined based on specific on-site sanitary survey results and the total dust concentration at the site, as tested by the relevant testing company.

#### Occupation high temperature [19]

Defined by the presence of a productive heat source and a WBGT ≥ 25℃.

#### Occupation noise [20]

Identified by the presence of harmful noise in the workplace, with workers exposed to an equivalent sound level ≥ 80 Bb(A) for 40 h per week or 8 h per day.

### Sample size calculation

In this investigation, the sample size calculation approach suggested by Richard [21] for clinical prediction models was applied.

1. To ensure that the model could correctly forecast the average of the outcome occurrences, ɵ was set at 29%, the total prevalence of NAFLD in China [22]. The error range δ is set to 0.05. At least 317 study cases were needed.
  1 $$\mathrm{n}={\left(\frac{1.96}{\updelta }\right)}^{2}\uptheta \left(1-\uptheta \right)$$
2. The mean absolute error MAPE was set at 0.05, and the predictor variable P was approximately 10 to adjust for the smallest mean error in the predicted values for all participants. At least 453 study cases were needed.
  2 $$\mathrm{n}=\mathrm{exp}\left(\frac{-0.508+0.259\mathrm{ln}\left(\varnothing \right)+0.504\mathrm{ln}\left(P\right)-\mathrm{ln}\left(MAPE\right)}{0.544}\right)$$
3. Based on a 29% prevalence outcome share, the estimated maxR_CS_^2^ was set at 0.45. The R_CS_^2^ value was set as 0.07 to ensure that the model could elucidate 15% of the variation. To prevent overfitting of the model and ensure an expected contraction rate of 10%, S was set to 0.9. The study variable *P* is approximately 10. At least 1250 study cases were needed.
  3 $$\mathrm{n}=\frac{P}{\left(S-1\right)\mathrm{ln}\left(1-\frac{{R}_{CS}^{2}}{S}\right)}$$
4. According to the above settings, S was calculated to be 0.756. To ensure minimal differences between the developed model and R_CS_^2^ optimally adjusted values, at least 435 study cases were needed.
  4 $$\mathrm{s}=\frac{{R}_{CS}^{2}}{{R}_{CS}^{2}+\mathrm{\delta max}{R}_{CS}^{2}}$$
  5 $$\mathrm{n}=\frac{P}{\left(S-1\right)\mathrm{ln}\left(1-\frac{{R}_{CS}^{2}}{S}\right)}$$

A minimum sample of 1250 cases was calculated to build the model. The study covered 3328 patients in all.

### Construction of the model

The modeling data were randomly partitioned into a training set and a test set in a 7:3 ratio. The model underwent training and parameter optimization based on the training set. The proficiency of the model was evaluated using sensitivity, precision, accuracy, Brier score, F1 score, log loss, ROC curve, and calibration curve, as demonstrated in Additional files 1 and 2.

The logistic regression model was built employing'sklearn.linear_mode', and parameter C was calibrated using a fivefold cross-validated logistic regression. The random forest model was developed using'sklearn. ensemble', and a grid search (cv = 5) was utilized to adjust the parameters, including criterion, max_depth, max_features, min_samples_leaf, min_samples_split, and n_estimators. The XGBoost model was constructed utilizing 'xgboost', and parameters such as learning_rate, max_depth, and n_estimators were fine-tuned using a grid search (cv = 5), as elucidated in Additional files 3, 4, 5 and 6.

### Statistical analysis

An Excel database was assembled according to the outcomes of the physical examination and questionnaire, aiming to identify risk factors and develop an assessment model. Count data were denoted using ratios or rates. The χ^2^ test was employed for comparisons between the two groups. Unconditional logistic regression facilitated the execution of multifactorial analysis. A two-sided test was applied with a significance level of 0.05. The correlation analyses pertinent to this study were carried out using the statistical software SPSS 25.0 and Python 3.8.

### Quality control

All researchers participating in the study underwent comprehensive training. The inclusion of study participants in the study was conducted strictly by the inclusion and exclusion criteria. Data entry was double-checked. The accuracy of the data was confirmed through manual, computerized, and logical error checks on the inputted information. For data analysis, the datasets were randomly partitioned into training and test sets.

## Research findings

### Single-factor analysis

The study ultimately encompassed 3328 steel workers, comprising 2908 males and 420 females, primarily within the age range of 40–49 years. The prevalence of NAFLD in this population was 35.64%, and over half of the workers were classified as overweight or obese.

Univariate analysis was performed on basic demographic attributes, behavioral lifestyles, occupational factor exposure, and liver function biochemical indicators of steelworkers. The results suggested that factors such as age, sex, BMI, hypertension, coronary heart disease, diabetes, hyperuricemia, dyslipidemia, smoking habits, DASH diet score, shift work, exposure to high temperature and dust, and ALT, AST and GGT levels were significantly correlated with the prevalence of NAFLD (*P* < 0.05) (Tables 1, 2, 3 and 4).

**Table 1:** Comparison of basic conditions aof steelworkers with and without NAFLD

Basic conditions		Non-NAFLD	NAFLD	*χ*^*2*^	*P*
Age (Year)				15.928	0.001
	< 30	121	50		
	30 ~ 39	537	368		
	40 ~ 49	916	487		
	50 ~ 60	568	281		
Sex				66.246	< 0.001
	Male	1797	1111		
	Female	345	75		
Education				1.343	0.511
	Elementary and below	18	14		
	Middle or high school	1584	862		
	College or above	540	310		
Marital status				1.951	0.377
	Unmarried	95	42		
	Married or remarried	1999	1113		
	Divorced or widowed	48	31		
Nation				1.166	0.280
	Han	2100	1156		
	Other	42	30		
Monthly income per capita of the household (Yuan)				0.985	0.611
	< 1500	656	375		
	1500 ~ 2500	923	490		
	> 2500	563	321		
BMI (kg/m)^2^				787.667	< 0.001
	< 24	1122	128		
	24 ~ 27.9	835	552		
	≥ 28	185	506		
Family history of hypertension				2.143	0.134
	No	1515	810		
	Yes	627	376		
Family history of dyslipidemia				0.058	0.810
	No	2043	1129		
	Yes	99	57		
Hypertension				59.306	< 0.001
	No	1928	955		
	Yes	214	231		
Coronary heart disease				4.578	0.032
	No	2121	1164		
	Yes	21	22		
Diabetes				8.621	0.003
	No	2067	1119		
	Yes	75	67		
Hyperuricemia				233.107	< 0.001
	No	1664	617		
	Yes	478	569		
Dyslipidemia				81.931	< 0.001
	No	2061	1044		
	Yes	81	142

**Table 2:** Comparison of the behavioral lifestyle of steelworkers with and without NAFLD

Behavioral lifestyle		Non-NAFLD	NAFLD	*χ*^*2*^	*P*
Smoking status				27.369	< 0.001
	Never smoking	1196	551		
	Quit smoking	99	60		
	Smoking	867	575		
DASH score				4.812	0.028
	< 25	795	486		
	≥ 25	1347	700		
Physical activity				0.057	0.972
	Mild	72	41		
	Moderate	166	94		
	Severe	1904	1051

**Table 3:** Comparison of occupational exposure factors of steelworkers with and without NAFLD

Factors		Non-NAFLD	NAFLD	χ^2^	*P*
Shift work				12.026	0.002
	Never shift	397	180		
	Once shift	395	188		
	Now shift	1350	818		
Occupation high temperature				7.923	0.005
	No	933	457		
	Yes	1209	729		
Occupation noise				0.909	0.340
	No	1061	567		
	Yes	1081	619		
Occupational dust				7.606	0.006
	No	1693	888		
	Yes	449	298

**Table 4:** Comparison of liver function index of steelworkers with and without NAFLD

Liver function index		Non-NAFLD	NAFLD	*χ*^*2*^	*P*
ALT (U/L)	≤ 40	2035	897	273.269	< 0.001
	> 40	107	289		
AST (U/L)	≤ 40	2120	1137	35.236	< 0.001
	> 40	22	49		
GGT(U/L)	≤ 50	1991	980	84.891	< 0.001
	> 50	151	206		
TBIL (μmol/L)	3.4 ~ 17.1	1734	954	2.548	0.280
	< 3.4	21	6		
	> 17.1	387	226		

### Multifactor analysis

To further delineate the factors influencing the prevalence of NAFLD among steelworkers, variables found to be statistically significant in the univariate analysis were subjected to a multifactorial logistic regression analysis. Detailed information regarding these variables, along with their assigned values, can be found in Table 5.

**Table 5:** Assignment table for variables

Variable	Variable Meaning	Assignment Method
Y	NAFLD	0 = No, 1 = Yes
X_1_	Age	1 = < 30, 2 = 30 ~ , 3 = 40 ~ , 4 = 50 ~ (year)
X_2_	Sex	1 = Male, 2 = Female
X_3_	BMI	1 = < 24, 2 = 24 ~ , 3 = 28 ~ (kg/m)^2^
X_4_	Smoking status	1 = Never smoking, 2 = Quit smoking, 3 = Smoking
X_5_	DASH score	1 = < 25, 2 = ≥ 25
X_6_	Hypertension	0 = No, 1 = Yes
X_7_	Coronary heart disease	0 = No, 1 = Yes
X_8_	Diabetes	0 = No, 1 = Yes
X_9_	Dyslipidemia	0 = No, 1 = Yes
X_10_	Hyperuricemia	0 = No, 1 = Yes
X_11_	Shift work	1 = Never shift, 2 = Once shift, 3 = Now shift
X_12_	Occupation high temperature	0 = No, 1 = Yes
X_13_	Occupational dust	0 = No, 1 = Yes
X_14_	ALT	0 = Normal, 1 = Abnormal
X_15_	AST	0 = Normal, 1 = Abnormal
X_16_	GGT	1 = Normal, 2 = Low, 3 = High

Prior to executing the multifactor logistic regression analysis, the incorporated factors underwent diagnosis for multicollinearity, as delineated in Table 6. The absence of collinearity between variables is indicated when tolerance > 0.1 and VIF < 10. According to the analysis outcomes, there was no observed correlation between the variables, thereby justifying the feasibility of linear analysis.

**Table 6:** Multicollinearity diagnosis of the study variables

Variable	Tolerance	VIF
Age	0.914	1.094
Sex	0.840	1.190
BMI	0.874	1.144
Smoking status	0.878	1.139
DASH score	0.975	1.026
Hypertension	0.901	1.110
Coronary heart disease	0.978	1.023
Diabetes	0.942	1.062
Dyslipidemia	0.930	1.075
Shift work	0.961	1.041
Occupation high temperature	0.861	1.162
Occupational dust	0.912	1.096
Hyperuricemia	0.897	1.115
ALT	0.775	1.290
AST	0.857	1.167
GGT	0.872	1.147

The results of the multifactorial analysis revealed that sex, BMI, hyperuricemia, dyslipidemia, occupational dust exposure, and ALT were associated risk factors for NAFLD in steelworkers (*P* < 0.05). Notably, female sex emerged as a protective factor against NAFLD, as illustrated in Table 7.

**Table 7:** Multivariate logistics regression analysis of risk factors for NAFLD in steel workers

Variable	B	S.E	Wald	df	Sig	Exp(B)	95% C.I. for Exp (B)	
							Lower	Upper
Age (Year)								
< 30			3.420	3	0.331			
30 ~	0.342	0.224	2.329	1	0.127	1.408	0.907	2.185
40 ~	0.234	0.221	1.122	1	0.289	1.263	0.820	1.946
50 ~	0.166	0.228	0.533	1	0.465	1.181	0.755	1.847
Sex	-0.397	0.165	5.814	1	0.016	0.672	0.487	0.928
BMI (kg/m2)								
< 24			447.232	2	< 0.001			
24 ~	1.604	0.113	200.331	1	< 0.001	4.971	3.981	6.207
28 ~	2.827	0.134	445.832	1	< 0.001	16.887	12.99	21.953
Hypertension	0.210	0.127	2.732	1	0.098	1.233	0.962	1.581
Coronary heart disease	0.332	0.368	0.813	1	0.367	1.393	0.677	2.867
Diabetes	0.368	0.212	3.026	1	0.082	1.445	0.954	2.187
Hyperuricemia	0.753	0.093	65.489	1	< 0.001	2.124	1.770	2.548
Dyslipidemia	0.839	0.179	21.985	1	< 0.001	2.315	1.630	3.288
Smoking status								
Never smoking			1.821	2	0.402			
Quit smoking	-0.130	0.207	0.394	1	0.530	0.878	0.585	1.318
Smoking	0.095	0.094	1.024	1	0.312	1.100	0.915	1.323
DASH score	-0.058	0.090	0.415	1	0.520	0.943	0.790	1.126
Shift work								
Never shift			3.151	2	0.207			
Once shift	0.021	0.153	0.018	1	0.892	1.021	0.757	1.377
Now shift	0.177	0.123	2.062	1	0.151	1.194	0.937	1.520
Occupation high temperature	-0.140	0.096	2.140	1	0.144	0.869	0.720	1.049
Occupational dust	0.226	0.108	4.355	1	0.037	1.254	1.014	1.551
ALT	1.289	0.150	73.914	1	< 0.001	3.629	2.705	4.869
AST	-0.198	0.343	0.333	1	0.564	0.820	0.419	1.607
GGT	0.272	0.145	3.527	1	0.060	1.312	0.988	1.743

### Risk assessment model for steelworkers

The multifactorial analysis results, when combined with a review of pertinent literature, culminated in the selection of nine factors to serve as variables within the assessment model. These included sex, BMI, hyperuricemia, dyslipidemia, occupational dust exposure, ALT, GGT, hypertension, and diabetes mellitus.

**Fig. 1:**
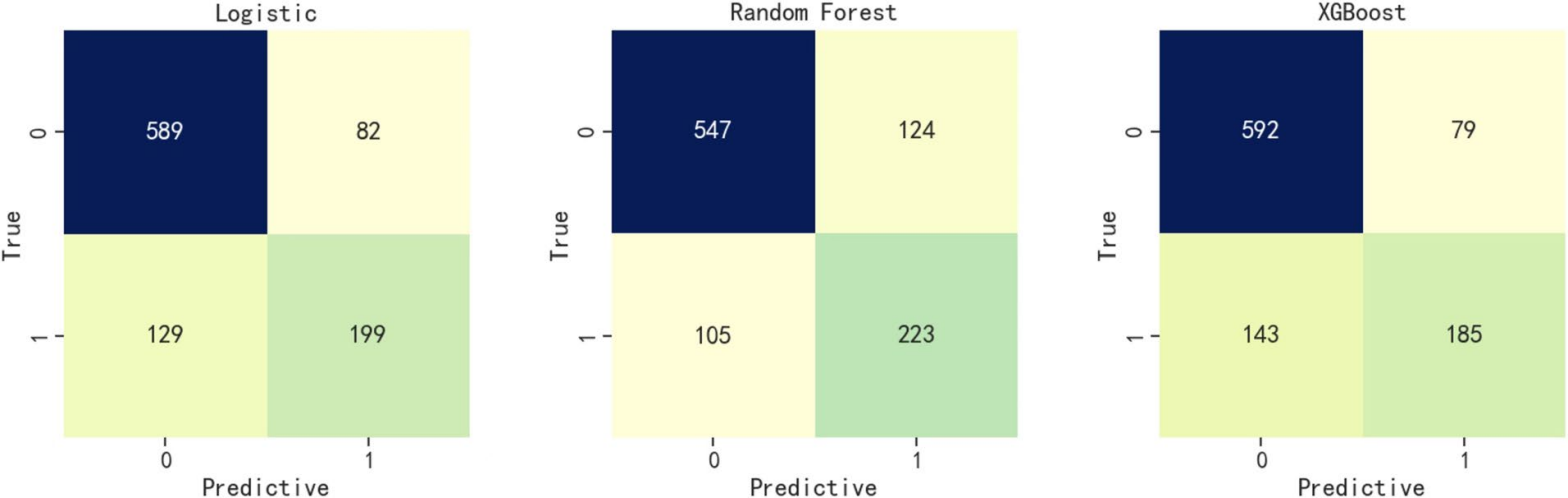
Confusion matrix of three models (True-0: actual non-NAFLD, True-1: actual NAFLD, Predictive-0: predicted non-NAFLD, Predictive-1: predictive NAFLD)

A total of 2329 individuals, equating to 70% of the participants, comprised the training set, while the test set included 999 individuals or 30% of the total participants. The projected and actual results for each model were juxtaposed to construct the corresponding confusion matrices. The efficacy of the three models used for assessing NAFLD in steel workers is depicted in Fig. 1.

**Fig. 2:**
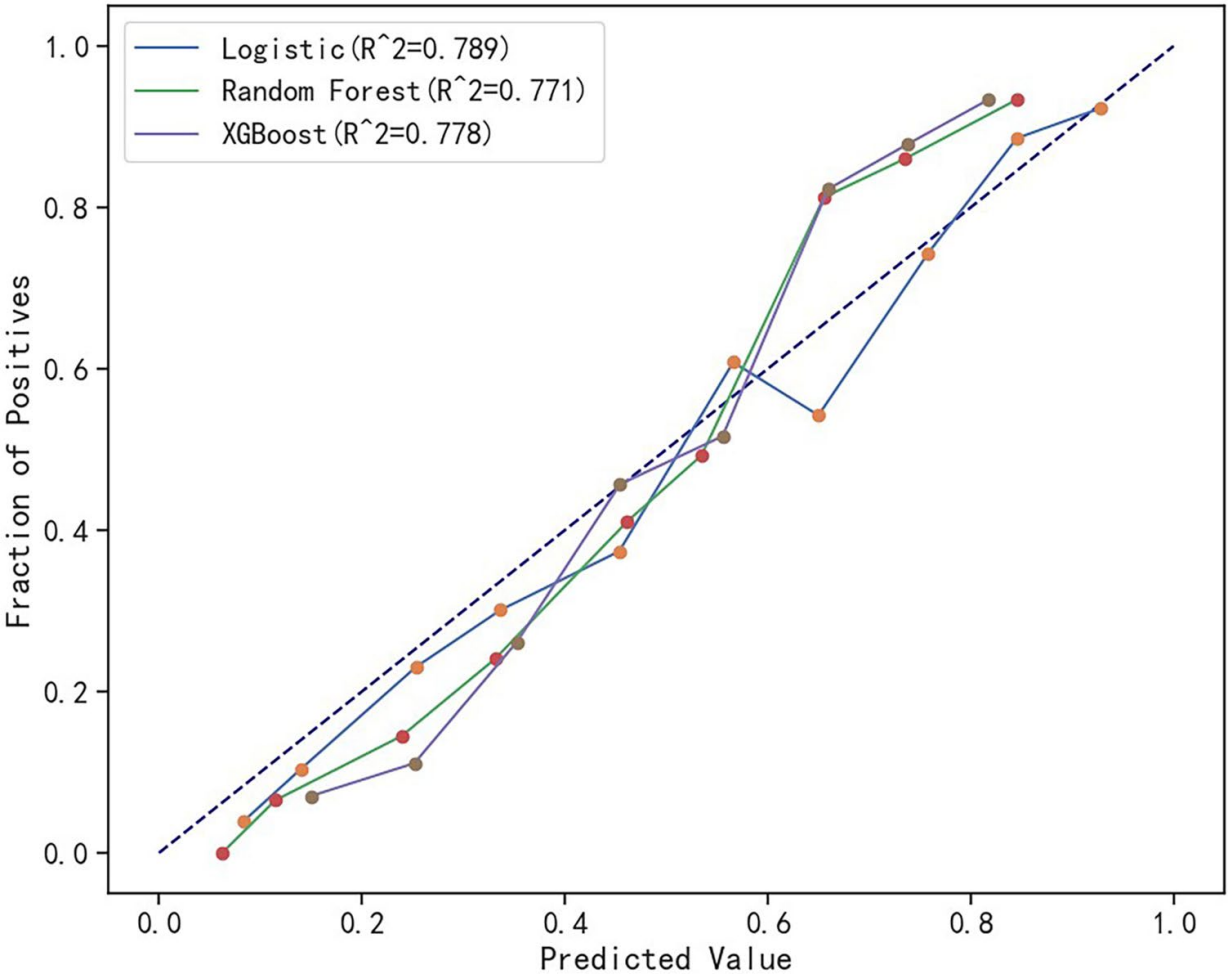
Calibration curves of the three models

**Fig. 3:**
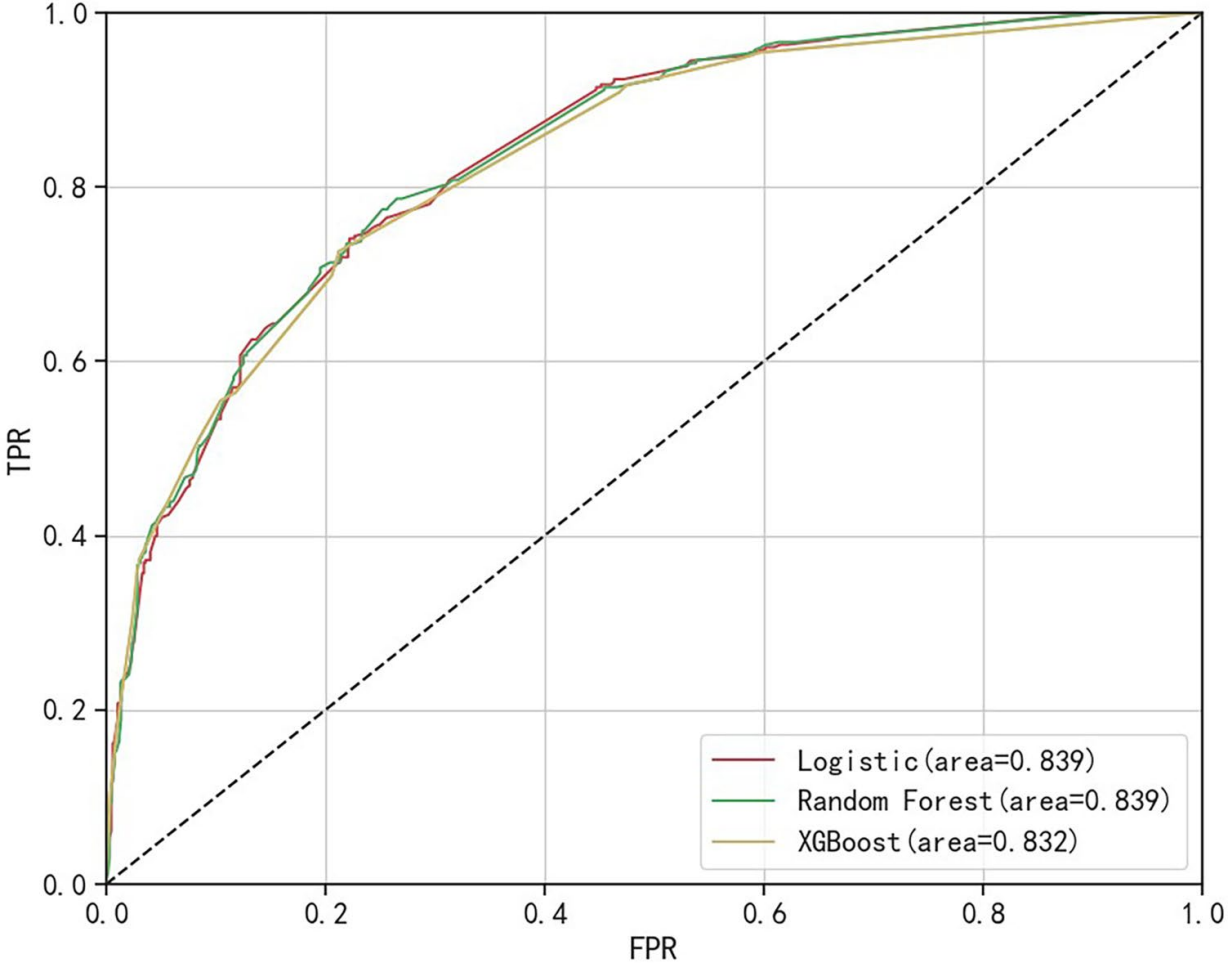
ROC curves of the three models

The comparative sensitivity for logistic regression, random forest, and XGBoost models was established at 0.607, 0.680, and 0.564, respectively. In terms of precision, the models scored 0.708, 0.643, and 0.701, respectively. The recorded accuracy was 0.789 for logistic regression, 0.771 for random forest, and 0.778 for XGBoost, with AUC results of 0.839, 0.839, and 0.832, respectively. The Brier score results stood at 0.150, 0.153, and 0.155 for each model in the same order, and the F1 score was measured at 0.654, 0.661, and 0.625. The log loss data came in at 0.460, 0.471, and 0.481, respectively. The R^2^ results for the models were 0.789, 0.771, and 0.778. All three models demonstrated good calibration, with their calibration curves oscillating around the diagonal. In terms of discrimination and calibration, the logistic regression model exhibited no significant deviation from the random forest and XGBoost models. Details are available in Table 8, Figs. 2, and 3.

**Table 8:** Comparison of the predictive performance of the three models

Evaluation index	Logistic	Random Forest	XGBoost
Sensitivity	0.607	0.680	0.564
Precision	0.708	0.643	0.701
Accuracy	0.789	0.771	0.778
AUC	0.839	0.839	0.832
Brier	0.150	0.153	0.155
F1	0.654	0.661	0.625
Log loss	0.460	0.471	0.481
R^2^	0.789	0.771	0.778

A risk assessment model for NAFLD in steelworkers was constructed based on logistic regression, and the details of the model are shown in Table 9. The equation of the logistic regression model for NAFLD risk assessment is shown as follows:

**Table 9:** Logistic regression model of NAFLD for steelworkers

Variable	B	S.E	Wald	df	Sig	Exp(B)	95% C.I. for Exp (B)	
							Lower	Upper
Sex (X _Sex_)	-0.398	0.153	6.718	1	0.010	0.672	0.497	0.908
BMI (kg/m2)								
< 24			451.122	2	< 0.001			
24 ~ (X _BMI1_)	1.598	0.113	200.929	1	< 0.001	4.944	3.964	6.166
28 ~ (X _BMI2_)	2.824	0.133	449.833	1	< 0.001	16.841	12.973	21.862
Hypertension (X _HTN_)	0.200	0.124	2.592	1	0.107	1.221	0.958	1.557
Diabetes (X _DM_)	0.345	0.209	2.733	1	0.098	1.412	0.938	2.126
Occupational dust (X _Dust_)	0.183	0.103	3.152	1	0.076	1.201	0.981	1.469
Hyperuricemia (X _HUA_)	0.763	0.092	69.233	1	< 0.001	2.145	1.792	2.567
ALT (X _ALT_)	1.276	0.142	81.131	1	< 0.001	3.581	2.713	4.727
GGT (X _GGT_)	0.300	0.143	4.399	1	0.036	1.350	1.020	1.786
Dyslipidemia (X _Dyslipidemia_)	0.833	0.177	22.025	1	< 0.001	2.299	1.624	3.256
constant	-2.570	0.110	549.931	1	0	0.077		

$$\mathrm{Logit}(P)= -2.57+ 1.598{\mathrm{X}}_{\mathrm{BMI}1} + 2.824{\mathrm{X}}_{\mathrm{BMI}2} + 0.2{\mathrm{X}}_{\mathrm{HTN}} + 0.345{\mathrm{X}}_{\mathrm{DM}} + 0.183{\mathrm{X}}_{\mathrm{Dust}} + 0.763{\mathrm{X}}_{\mathrm{HUA}} + 1.276{\mathrm{X}}_{\mathrm{ALT}} + 0.3{\mathrm{X}}_{\mathrm{GGT}} + 0.883{\mathrm{X}}_{\mathrm{Dyslipidemia}}-0.398{\mathrm{X}}_{\mathrm{Sex}}$$

**Fig. 4:**
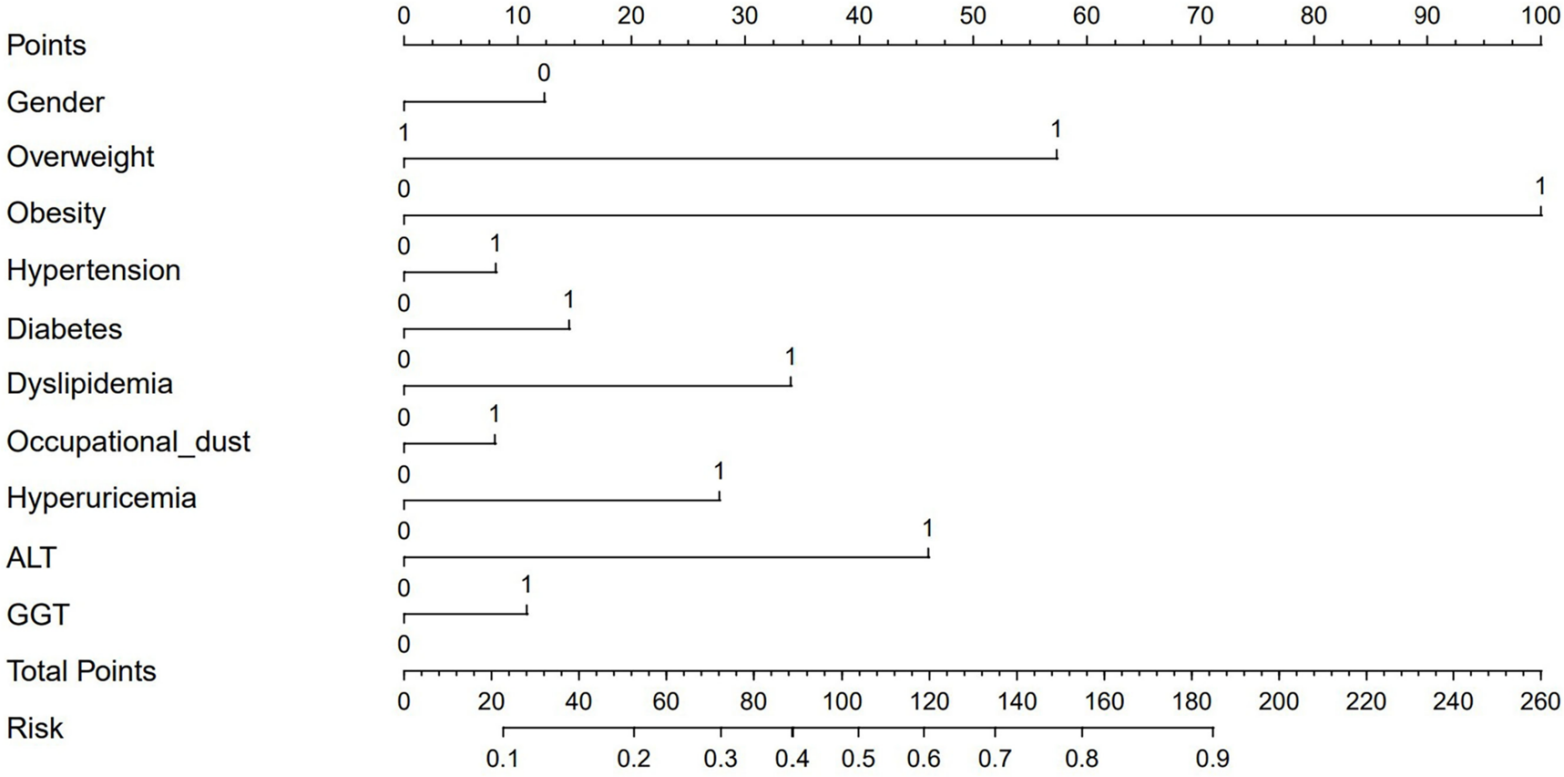
Nomogram for risk assessment of NAFLD in steelworkers (Sex-0: Male, Sex-1: Female, ALT-0: Normal, ALT-1: Abnormal, GGT-0: Normal, GGT-1: Abnormal, Other Indicators-0: No, Other Indicators-1: Yes)

**Fig. 5:**
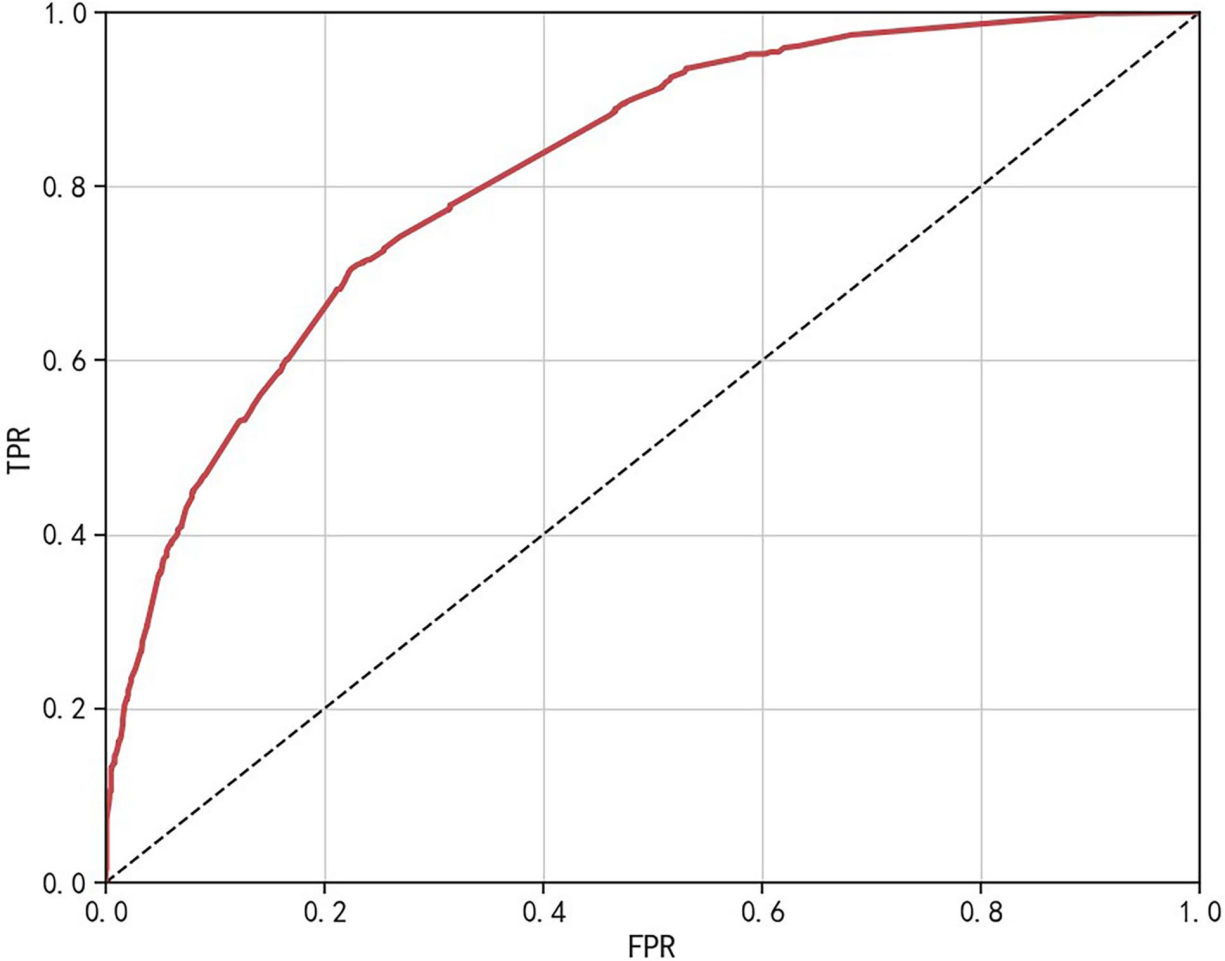
ROC curve for screening NAFLD risk scores

In this study, a nomogram for assessing the risk of NAFLD in steelworkers was derived from the logistic regression model, as depicted in Fig. 4. Using this nomogram, a random selection of 2329 study participants was scored, and an ROC curve was subsequently plotted using the individual scores in correlation with the prevalence of NAFLD, as illustrated in Fig. 5. At the optimal Jordan Index value of 0.481, sensitivity and specificity were measured at 0.705 and 0.776, respectively, resulting in an optimal cutoff score of 86. Therefore, it was determined that workers with scores below 86 fell into the low-risk category, while those with scores of 86 or above were categorized as high-risk individuals.

**Fig. 6:**
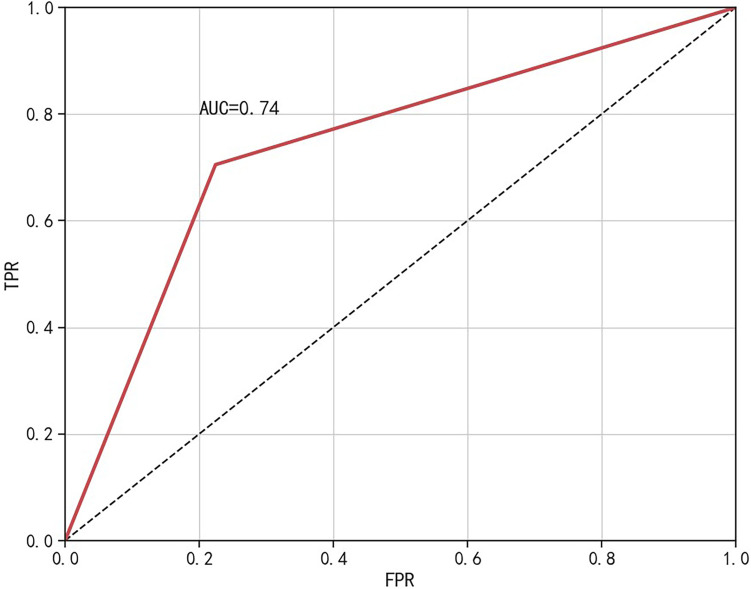
ROC curve for establishing the NAFLD risk scoring system

The resulting classifications revealed a significant disparity in the prevalence of NAFLD between the high-risk and low-risk groups. Among the low-risk individuals, 18.15% were identified with NAFLD, compared to 64.71% within the high-risk category. The risk scoring system demonstrated effective risk stratification capabilities, with an accuracy of 74.97% and an area under the curve (AUC) of 0.740, as detailed in Table 10 and Fig. 6.

**Table 10:** Classification results of the NAFLD disease risk scoring system

Risk stratification	Score	Total	NAFLD(n%)		χ^2^	*P*
			No	Yes		
Low risk	< 86	1394	1141(81.85)	253(18.15)	521.324	< 0.001
High risk	≥ 86	935	330(35.29)	605(64.71)

## Discussion

In this study, the prevalence of NAFLD among steelworkers was found to be 35.64%, surpassing the general population prevalence rate of 32.9% [22]. Male workers, with a prevalence rate of 38.2%, appeared to be more susceptible to NAFLD than their female counterparts, whose prevalence rate was 17.86%. This pattern was observed even among children and adolescents, where males demonstrated a higher NAFLD prevalence than females (17.86%) [23, 24]. These findings suggest that female sex acts as a protective factor against NAFLD. This can be attributed to the role of estrogen, which is known to encourage subcutaneous fat deposition and inhibit lipolysis, thereby reducing the influx of free fatty acids (FFAs) to the liver. Moreover, estrogen impedes diet-induced de novo lipogenesis, thereby promoting higher hepatic metabolic activity in females [25]. Studies conducted on diet-induced NAFLD in mice have revealed more pronounced liver steatosis in males than in females. Furthermore, the upregulation of fibroblast growth factor 21 (FGF21) expression in female liver tissues led to gender-specific browning of gonadal white adipose tissue to some extent, reinforcing the notion that NAFLD is a sexually dimorphic disease [26].

In the current study, being overweight or obese was identified as a significant contributing factor to NAFLD, corroborating previous research [27]. The liver, an essential organ in lipid and glucose metabolism, is particularly vulnerable to the effects of obesity [28]. Investigations have revealed upregulated expression of FTO (fat mass and obesity-associated gene), a known metabolic disease predictor, in both NAFLD patients and animal models and that abnormal hepatic signaling activity of FTO was associated with impaired metabolism in NAFLD [29]. This substantiates the notion that overweight or obesity amplifies the risk of NAFLD at the molecular level. Moreover, individuals with dyslipidemia demonstrated a higher propensity toward NAFLD development in this study. Research by Tsuneto et al. established a significant link between the development of NAFLD, hypertriglyceridemia, and obesity [30]. Even in the nonobese population, elevated LDL-C levels independently influenced the development of NAFLD [31]. Dyslipidemia in NAFLD patients is characterized by heightened TG and LDL-C levels and reduced HDL-C concentrations, a condition known as atherogenic dyslipidemia, leading to an increased risk of CVD morbidity and mortality [32]. Therefore, proactive and effective lipid-lowering strategies such as maintaining a healthy diet and adequate physical activity can not only aid in managing the population's prevalence of NAFLD but also prevent the onset of CVD, offering substantial health benefits.

Hyperuricemia (HUA) has been associated with mitochondrial dysfunction and reactive oxygen species production, and it can activate AP-1 via the c-Jun N-terminal kinase (JNK) pathway. This upregulates the expression of adipogenic genes, thereby influencing the progression of NAFLD [33]. NAFLD is observed more frequently in individuals with HUA than in those with normal blood uric acid levels [34]. Notably, within the obese population, for every unit increase in blood uric acid, the controlled attenuation parameter (CAP) for liver fat escalates by 14 dB/m, implying that uric acid levels serve as a vital metabolic screening tool for NAFLD [35]. In this investigation, a significant correlation was observed between the prevalence of NAFLD and HUA among steelworkers. Given that over half (52.44%) of the workers were overweight or obese, it is essential to place an increased emphasis on monitoring their blood uric acid levels.

This study identified an elevated risk of NAFLD among workers exposed to occupational dust. Dust, as a prevalent factor impacting the health of occupational groups, poses a significant risk for cardiovascular diseases such as hypertension and atherosclerosis [36]. Additionally, hypertension can independently affect fatty liver, suggesting that dust may be associated with NAFLD [37]. A study conducted on the World Trade Centre General Responder Cohort (WTC GRC) in the USA found dust exposure to be a potent independent predictor of hepatic steatosis [38]. However, no additive or synergistic effect of dust was discovered in the investigation of noise and fatty liver [39]. Given the limited research on the impact of dust on NAFLD, further investigation in this area is warranted. Furthermore, this study found abnormal ALT levels to be a significant risk factor for NAFLD, corroborating the findings of Shao et al. [40]. After adjusting for sex, ALT was found to be a reliable predictor for the prevalence of NAFLD in both males and females, underscoring the importance of ALT in the diagnosis of NAFLD [24].

Previous research indicates that individuals with diabetes exhibit an increased risk of progressing to advanced fibrosis [41]. NAFLD further contributes to the development of diabetes by exacerbating both hepatic and peripheral insulin resistance and prompting the systemic release of proinflammatory cytokines and hepatic factors [42]. These findings underscore a robust association between diabetes and NAFLD. In the current study, however, the influence of diabetes mellitus on the prevalence of NAFLD was not statistically significant, which could potentially be attributed to the unique characteristics of the steelworker population. Compared to the general population, steelworkers undergoing an induction medical examination generally exhibit a superior physical condition, which could consequently reduce their susceptibility to certain diseases.

As science and technology advance, accompanied by the increasing digitization of information, machine learning has become progressively influential in the medical field. In pain medicine, support vector classification (SVC) and convolutional neural network (CNN) algorithms have been extensively employed in research on pain assessment and diagnosis [43]. Models such as random forest and XGBoost have played pivotal roles in predicting the prognosis of gynecological diseases, specifically cervical cancer, and in the diagnosis of ovarian cancer [44]. The random forest algorithm, proposed in 2001, stands as a representative of ensemble algorithms. It enhances prediction accuracy without necessitating a substantial increase in computing power and exhibits robust performance amidst random disturbances, regardless of outliers [45]. In contrast, the XGBoost algorithm, introduced in 2016, embodies an efficient implementation of the gradient boosting concept, ensuring high computational efficiency while maintaining effective overfitting prevention attributes [46]. Despite being a classical prediction method, the logistic regression model has demonstrated commendable prediction outcomes in forecasting short-term asthma exacerbations when compared to other machine learning models [47]. The process of screening and modeling for disease-specific risk assessment facilitates earlier detection and treatment of diseases, thereby aiding in efficient disease diagnosis and management.

Upon reviewing the literature and the outcomes of the factor analyses, nine variables were incorporated into the assessment model analysis. The results indicated that the area under the curve (AUC) values for logistic regression, random forest, and XGBoost were 0.839, 0.839, and 0.832, respectively, with no substantial differences across other indicators. This suggests that all three models demonstrate commendable assessment performance. However, in practical implementations, it is imperative to consider both the interpretability and performance of the risk assessment model [48]. The logistic regression model, as a conventional modeling procedure, not only allows for the screening of potential influential factors of a disease but also provides a quantitative interpretation of the impact of each variable. Evaluating the significance of each factor in differential diagnosis using odds ratio (OR) values enhances the model's versatility in application [49, 50]. Compared to the random forest and XGBoost models, the logistic regression model can depict the prediction process in the form of exceptionally straightforward equations, resulting in greater transparency and interpretability, making it more apt for use in the medical domain. Hence, the logistic regression model was ultimately selected for this study to perform a risk assessment of nonalcoholic fatty liver disease (NAFLD) in steelworkers. The evaluation of the NAFLD risk scoring system revealed an accuracy rate of 74.97% and an AUC of 0.740, demonstrating effective risk identification and facilitating the advancement of early prevention and treatment of high-risk workers.

### Study strengths and limitations

This study was grounded on the Beijing-Tianjin-Hebei Occupational Cohort, which ensured a high level of integrity and reliability in the results. To guarantee superior performance metrics, the model parameters were refined using fivefold cross-validation and a grid search. Moreover, we proposed a cutoff value for the nonalcoholic fatty liver disease (NAFLD) risk score among steelworkers, thereby facilitating targeted NAFLD risk stratification among this workforce.

Nonetheless, several limitations were inherent to this research. First, the study's outcomes were predicated upon a comparison between logistic regression, random forest, and XGBoost assessment models without investigating the impact of other potential models. Second, the research was conducted specifically within a steelworker population; thus, its findings cannot be generalized to the broader population. Third, the unique nature of the study cohort necessitated an internal validation approach, restricting our ability to evaluate the model's predictive power for NAFLD prevalence among other steelworker groups.

## Conclusion

Sex, BMI, dyslipidemia, hyperuricemia, occupational dust exposure, and ALT were influential NAFLD risk factors among steelworkers. For risk assessment studies of NAFLD in this demographic, the traditional logistic regression model exhibited comparable excellence to the random forest and XGBoost models. The optimal cutoff value for risk assessment was established at 86. This study offers clinicians a straightforward visual risk rating approach to evaluate the likelihood of NAFLD in steelworkers, helping to identify and intervene early in those at risk.

### Supplementary Information

**Additional file 1. **

**Additional file 2. **

**Additional file 3. **

**Additional file 4. **

**Additional file 5. **

**Additional file 6. **

## Data Availability

The datasets used and analyzed during the current study are available from the corresponding author on reasonable request.

## Abbreviations

NAFLD: Nonalcoholic fatty liver disease
TC: Total cholesterol
TG: Triglyceride
HDL-C: High-density lipoprotein cholesterol
LDL-C: Low-density lipoprotein cholesterol
ALT: Alanine aminotransferase
AST: Aspartate aminotransferase
GGT: Glutamyl transpeptidase
T-bill: Total bilirubin
SBP: Systolic blood pressure
DBP: Diastolic blood pressure
FFAs: Free fatty acids
FGF21: Fibroblast growth factor 21
CVD: Cardiovascular disease
HUA: Hyperuricemia
